# Time-to-hepatitis C treatment initiation among people who inject drugs in Melbourne, Australia

**DOI:** 10.1017/S0950268823000675

**Published:** 2023-05-09

**Authors:** Phyo T. Z. Aung, Tim Spelman, Anna L. Wilkinson, Paul M. Dietze, Mark A. Stoové, Margaret E. Hellard

**Affiliations:** 1 Disease Elimination Program, Burnet Institute, Melbourne, VIC, Australia; 2 Department of Epidemiology and Preventive Medicine, Monash University, Melbourne, VIC, Australia; 3 Department of Infectious Diseases, Alfred Health & Monash University, Melbourne, VIC, Australia; 4 National Drug research Institute and Enable Institute, Curtin University, Melbourne, VIC, Australia

**Keywords:** Direct-acting antivirals, hepatitis C, people who inject drugs, time-to-treatment

## Abstract

This study aims to understand the time-to-treatment initiation pre and post DAA access to inform strategies to improve HCV care. The data for our study were derived from the SuperMIX cohort study of people who inject drugs in Melbourne, Australia. Time-to-event analysis using Weibull accelerated failure time was performed for data collected between 2009 and 2021, among a cohort of HCV-positive participants. Among 223 participants who tested positive for active hepatitis C infection, 102 people (45.7%) reported treatment initiation, with a median time-to-treatment of 7 years. However, the median time-to-treatment reduced to 2.3 years for those tested positive after 2016. The study found that treatment with Opioid Agonist Therapy (TR 0.7, 95% CI 0.6–0.9), engagement with health or social services (TR 0.7, 95% CI 0.6–0.9), and having a first positive HCV RNA test after March 2016 (TR 0.3, 95% CI 0.2–0.3) were associated with a reduced time-to-treatment initiation. The study highlights the need for strategies to improve engagement with health services, including drug treatment services into routine HCV care to achieve timely treatment.

## Introduction

Injecting drug use is a major risk factor for the transmission of hepatitis C virus (HCV), with the sharing of needles, syringes, and other injecting equipment driving HCV transmission among people who inject drugs [1]. Among an approximate 75,000 people who inject drugs on a regular basis in Australia in a year, almost half were estimated to be living with HCV in 2016 [2–4]. In March 2016, Australia became one of the first countries to fund broad access to HCV treatment by listing new oral direct-acting antiviral medications (DAAs) on the Pharmaceutical Benefits Scheme (PBS), Australia’s system for medicine subsidy under its universal health care coverage [5–8]. A key feature of this PBS listing was allowing widespread access to DAAs for all people living with chronic HCV, including through prescribing by primary care clinicians and through prison health services. By the end of 2021, a total of 95,395 individuals (approximately 53% of the estimated number of people living with HCV at the end of 2015) initiated DAA treatment through the PBS [9]. Despite the initial surge in the treatment uptake in 2016, a steady downswing in the treatment numbers was observed in the subsequent years, meaning that Australia is at risk of missing the 2030 WHO HCV elimination targets (defined as a 90% reduction in new chronic infections and a 65% reduction in mortality, compared with the 2015 baseline) [5].

The decline in uptake of HCV treatment has been attributed to a combination of factors, including inadequate rates of testing, stigma experienced in health settings, and social and structural barriers such as poverty and homelessness [10–13]. Low treatment uptake may also be due to the poor integration of HCV care with other services catering to people who inject drugs and missed opportunities when people at risk of HCV present to health services [14]. For Australia to meet the WHO elimination targets, adapted models of care, such as integrated primary care services and peer-based models, are likely to be required [10].

Modelling and more recent empiric evidence suggest that treatment of HCV among people who inject drugs not only benefits the individual but can also have a treatment as prevention benefit [15]. Studies have also shown that DAA treatment scale-up was associated with reduced HCV incidence, especially in prison settings, highlighting the beneficial effect of HCV treatment as a prevention tool among people who inject drugs [16, 17]. Population-level HCV viremia is influenced both by the prevalence of chronic HCV and by the time individuals remain viremic, meaning the timely delivery or uptake of DAAs following HCV infection among people who inject drugs is likely to be particularly impactful. However, to date there is limited understanding as to how different socioeconomic factors influence the engagement in HCV care of people who inject drugs’ engagement in HCV care, in terms of time-to-treatment initiation specifically.

Further understanding of timely delivery or uptake of DAA is needed to inform strategies to minimise treatment delay. Our study aimed to describe trends in time-to-treatment initiation in a cohort of people who inject drugs in Melbourne, Australia, and identify the influence of personal, social, and drug use characteristics on time-to-treatment initiation, specifically on the speed of treatment uptake, i.e., whether these characteristics accelerate or decelerate treatment initiation.

## Methods

### Study design

This study is a time-to-event analysis of the Melbourne Injecting Drug User Cohort Study (SuperMIX), a prospective longitudinal study of people who inject drugs, designed to identify the trajectories of injecting drug use, along with a range of service use and health outcomes. We analysed longitudinal data on HCV treatment initiation from SuperMIX collected between November 2009 and March 2021 [18].

The SuperMIX cohort was approved by the Victorian Department of Health Human Research Ethics Committee (approval number: 28/13/17) and the Australian Institute of Health and Welfare Ethics Committee (approval number: EO2021/1/1241).

### Data source

The SuperMIX study involves baseline and follow-up interviews scheduled annually that collect data on demographic characteristics, current and past drug purchases and use, personal well-being, health service utilisation, criminal behaviour and interactions with justice systems, engagement in diagnostic testing, and treatment of blood-borne viruses including HCV. Since 2009, the study protocol has included venous blood sample collection by researchers for serological testing for HCV, hepatitis B virus, and HIV. HCV-specific blood tests performed at a reference laboratory were anti-HCV antibodies, HCV RNA, viral load, and genotyping. The SuperMIX cohort profile and additional information on study methodology have been described in detail elsewhere [19, 20].

The recruitment criteria for SuperMIX included being aged 18 and older, reporting having injected either heroin and/or amphetamine at least once a month for the six months prior to baseline interview, being willing to provide detailed contact information, having a valid Australian universal health care (Medicare) card number, and currently residing in Melbourne or the Greater Geelong region. Initially designed as a closed cohort, participants were recruited through a mix of respondent-driven and snowball sampling, and street-based outreach methods in two main waves, in 2008–2010 and in 2017–2019, and the design was changed to an open cohort from 2017. [18].

In SuperMIX, as there is no on-site clinician available to provide treatment, researchers attempt to contact participants and facilitate referral to HCV care following a positive HCV test (any of the tests listed above) reported to researchers by the reference laboratory as part of the study procedure. In the event of failed attempts to contact participants immediately following positive HCV results, participants were informed of their positive HCV result at their subsequent study visit.

### Study cohort selection

To study the characteristics associated with HCV treatment initiation, we included people whose blood results showed active HCV infection (i.e., the blood results were HCV RNA positive, and/or genotyping completed).

Participants enrolled from the SuperMIX cohort into a sub-study called the *Treatment as Prevention* study (TAP) were excluded to eliminate selection bias. TAP was a nurse-led study where a sample of people who inject drugs (including some SuperMIX participants) was invited to undergo treatment with DAAs. Detailed information on the TAP study is available elsewhere [21].

Participants who spontaneously cleared infection (defined as HCV RNA changed from positive to negative within six months following the first positive HCV RNA test) were excluded. If the RNA changed from positive to negative with more than six months between the tests but without evidence of treatment, we assumed either spontaneous clearance or undisclosed treatment. In these instances, given treatment history could not be ascertained, all participants who did not report treatment but showed evidence of resolved infection were also excluded.

To allow for an observation period where participants had the opportunity to access treatment and report to us whether treatment had or had not occurred, our analyses were limited to the participants who had at least one positive HCV RNA with a subsequent follow-up interview done not earlier than six months after the positive HCV RNA test date. For these participants, the analysis period commenced at the date of the first RNA-positive test recorded in the SuperMIX until the most recent interview for those not reporting treatment or estimated date of treatment (see section below) for those reporting treatment.

### Outcome measures

The primary outcome of this study was HCV treatment initiation. The outcome variable was derived from study interviews following the positive HCV RNA test, where the participants were asked if they had initiated HCV treatment since last seen. The interview schedule for HCV testing and treatment was developed using branching logic in the following order: ‘Have you had HCV test since last seen?’; ‘What was the test result?’ (if answered ‘yes’ to a HCV test); ‘Have you been offered HCV treatment?’ (if answered ‘yes’ to a positive result); ‘Have you initiated treatment?’ (if answered ‘yes’ to being offered treatment); ‘When did you initiate treatment?’ (if answered ‘yes’ to having initiated treatment). Participants were assigned their treatment status depending on whether they answered ‘Yes’ or ‘No’ to having initiated HCV treatment during the study analysis period. As only the month and the year of treatment initiation were collected during the interviews, the 15th day of the month (mid-month) was assigned as the treatment date.

**Figure 1.**
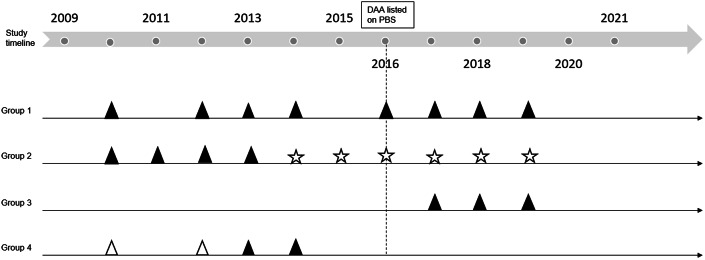
Schematic of HCV treatment missed opportunities. Dark triangles indicate positive HCV RNA test; white triangles represent negative HCV RNA test; stars denote interview visits without blood being drawn. Group 1: tested positive before 2016 and did not get treated despite remaining in the study after 2016; Group 2: tested positive up to a point before 2016 but did not have further HCV blood tests despite being followed up every year for interviews; Group 3: tested positive after 2016 and did not get treated; Group 4: left the study before 2016. Group 1 – 3 indicate missed opportunities for treatment in the DAA era

Missed opportunities for treatment, i.e., number of participants who missed out on treatment, were measured as secondary outcomes. A participant was considered a missed opportunity if they remained in the study after March 2016 and did not report treatment (Figure 1).

### Independent variables

A range of self-reported variables identified in the literature as being potentially related to HCV treatment uptake were selected. For participants who reported treatment initiation, time-varying variables were derived from the last interview that preceded the treatment initiation date, whilst for those who did not initiate treatment, time-varying variables were derived from the last available interview.

The variables analysed included demographic characteristics such as age, sex, Aboriginal and Torres Strait Islander status, country of birth, language spoken; socioeconomic characteristics such as education, employment, income, housing status, social support, history of incarceration; drug-use characteristics such as age of first injecting, duration of injecting career, recent injecting drug use; and service-related factors such as service attendance and accessing Opioid Agonist Therapy (OAT).

Most variables were dichotomised, with values listed in Table 1 and Supplementary material S1.

**Table 1.** Individual variables, treatment initiation and bivariate analyses

Individual variables			Treatment initiation (YES)		Treatment initiation (NO)		Bivariate analysis		
	N	%	n	%	n	%	TR	*P*-value	95% CI
*Overall*	223	100	102	45.7	121	54.3			
*Age at treatment or at interview if not treated (years)* ^a^									
20–30	26	11.7	12	11.8	14	11.6	Ref		
31–40	155	69.5	75	73.5	80	66.1	1.3	0.224	0.9–2.0
41–57	42	18.8	15	14.7	27	22.3	1.2	0.516	0.7–2.0
Missing	0								
*Sex*									
Male	156	70.0	71	69.6	85	70.3	Ref		
Female	67	30.0	31	30.4	36	29.7	1.1	0.480	0.8–1.5
Missing	0								
*Country of birth*									
Australia	183	82.1	84	82.4	99	81.8	Ref		
Other	40	17.9	18	17.7	22	18.2	1.1	0.714	0.7–1.5
Missing	0								
*Language spoken at home*									
English	192	86.1	88	86.3	104	86.0	Ref		
Other	31	13.9	14	13.7	17	14.0	1.2	0.324	0.8–1.8
Missing	0								
*Aboriginal and Torres Strait Islander*									
Yes	25	11.2	9	8.8	16	13.2	0.8	0.397	0.5–1.3
No	198	88.8	93	91.2	105	86.8	Ref		
Missing	0								
*Highest education level* ^a^									
Below year 10	52	23.3	26	25.5	26	21.5	Ref		
Year 10–11	73	32.7	27	26.5	46	38.0	1.3	0.160	0.9–1.9
Year 12 or higher	69	31.0	31	30.4	38	31.4	1.1	0.550	0.8–1.6
Missing	29	13.0	18	17.6	11	9.1			
*Employment* ^a^									
Unemployed	184	82.5	79	77.5	105	86.8	Ref		
Employed	39	17.5	23	22.5	16	13.2	0.9	0.469	0.6–1.2
Missing	0								
*Weekly income (AUD)* ^a^									
<$400	108	48.4	47	46.1	61	50.4	Ref		
≥$400	113	50.7	54	52.9	59	48.8	0.9	0.710	0.7–1.2
Missing	2	0.9	1	1.0	1	0.8			
*Accommodation* ^a^									
Stable	115	51.6	52	51.0	63	52.1	1.1	0.724	0.8–1.4
Unstable	102	45.7	47	46.1	55	45.5	Ref		
Missing	6	2.7	3	2.9	3	2.4			
*Social support* ^a^									
Yes	182	81.6	84	82.4	98	81.0	1.0	0.802	0.7–1.5
No	41	18.4	18	17.6	23	19.0	Ref		
Missing	0								
*Incarcerated within the 12 months* ^a^									
Yes	75	33.6	31	30.4	44	36.4	1.0	0.977	0.8–1.3
No	146	65.5	70	68.6	76	62.8	Ref		
Missing	2	0.9	1	1.0	1	0.8			
*Age of first injecting by median age (years)*									
<17	104	46.6	50	49.0	54	44.6	Ref		
> = 17	118	52.9	52	51.0	66	54.6	1.0	0.897	0.8–1.3
Missing	1	0.5	0	0	1	0.8			
*Duration of injecting drug use (years)* ^a^									
<18	96	43.0	46	45.1	50	41.3	Ref		
> = 18	127	57.0	56	54.9	71	58.7	**1.4**	**0.008**	**1.1–1.9**
Missing	0								
*Injecting drug use in the last one month* ^a^									
Yes	181	81.2	77	75.5	104	86.0	1.1	0.622	0.8–1.5
No	42	18.8	25	24.5	17	14.0	Ref		
Missing	0								
*OAT in the last one month* ^a^ ^,^^b^									
Yes	110	49.3	58	56.8	52	43.0	0.8	0.093	0.6–1.0
No	108	48.4	42	41.2	66	54.6	Ref		
Missing	5	2.2	2	2.0	3	2.4			
*Health or social service attendance in the last one month* ^a^ ^,^^b^									
Yes	165	74.0	84	82.4	81	66.9	**0.6**	**0.011**	**0.4–0.9**
No	58	26.0	18	17.6	40	33.1	Ref		
Missing	0								
*First HCV RNA–positive date*									
Before 2016	153	68.6	75	75.5	78	64.5	Ref		
After 2016	70	31.4	27	26.5	43	35.5	**0.3**	**<0.001**	**0.2–0.3**
Missing	0								

Note: Statistically significant values were highlighted in bold.

a Time varying variables were analysed at the most recent pre-treatment period.

b Service use may include attendances for OAT as well as other services listed in the Supplementary material S1, while OAT specifically represents treatment with methadone, suboxone, etc.

### Statistical methods

Descriptive analyses were undertaken (1) to describe the individual characteristics of the participants, (2) to describe the overall trend of treatment uptake, and (3) for the secondary outcomes, i.e., missed opportunities for treatment.

In the time-to-event analysis, Kaplan–Meier curves were used to visualise the time taken for a person to initiate treatment after the first positive HCV RNA test. To understand the influence of the socioeconomic characteristics on time-to-treatment initiation, multivariate analysis using accelerated failure time (AFT) model was employed. Parametric distributions such as Weibull, Exponential, Log-normal, and Log-logistic were considered and Weibull parametric model was selected based on Akaike’s Information Criterion (AIC) (Table 2). The exponential of the regression coefficient from the AFT model is referred to as time ratio (TR), which indicates whether the socioeconomic factors accelerate or decelerate the time-to-treatment initiation. A TR of less than 1 indicates that time-to-treatment is shortened whilst a TR greater than 1 means that time-to-treatment is lengthened.

**Table 2.** Comparison of AFT models

No	Model	Log-likelihood	AIC	BIC
1	Weibull	−159.3	344.7	388.7
2	Exponential	−191.8	407.6	448.2
3	Log-normal	−168.4	362.8	406.8
4	Log-logistic	−162.8	351.6	395.6

Prior to the multivariate analysis, bivariate analysis was performed on all the selected variables. The variables that reached statistical significance (*p* < 0.05) in bivariate analysis as well as clinically important variables from the literature which included age, sex, employment, housing status, social support, history of incarceration, and OAT were used to fit the multivariate AFT model [3, 13, 22, 23]. In addition, as the introduction of DAAs likely influenced the time between diagnosis and treatment, the first positive HCV RNA test before or after 2016 (when the universal access to DAAs commenced) was included as a confounder in the multivariate model. Listwise deletion methods were applied to handle missing data.

Data were analysed using Stata software version 17 (Stata Corporation, College Station, TX, USA) and Excel.

## Results

### Study population

**Figure 2.**
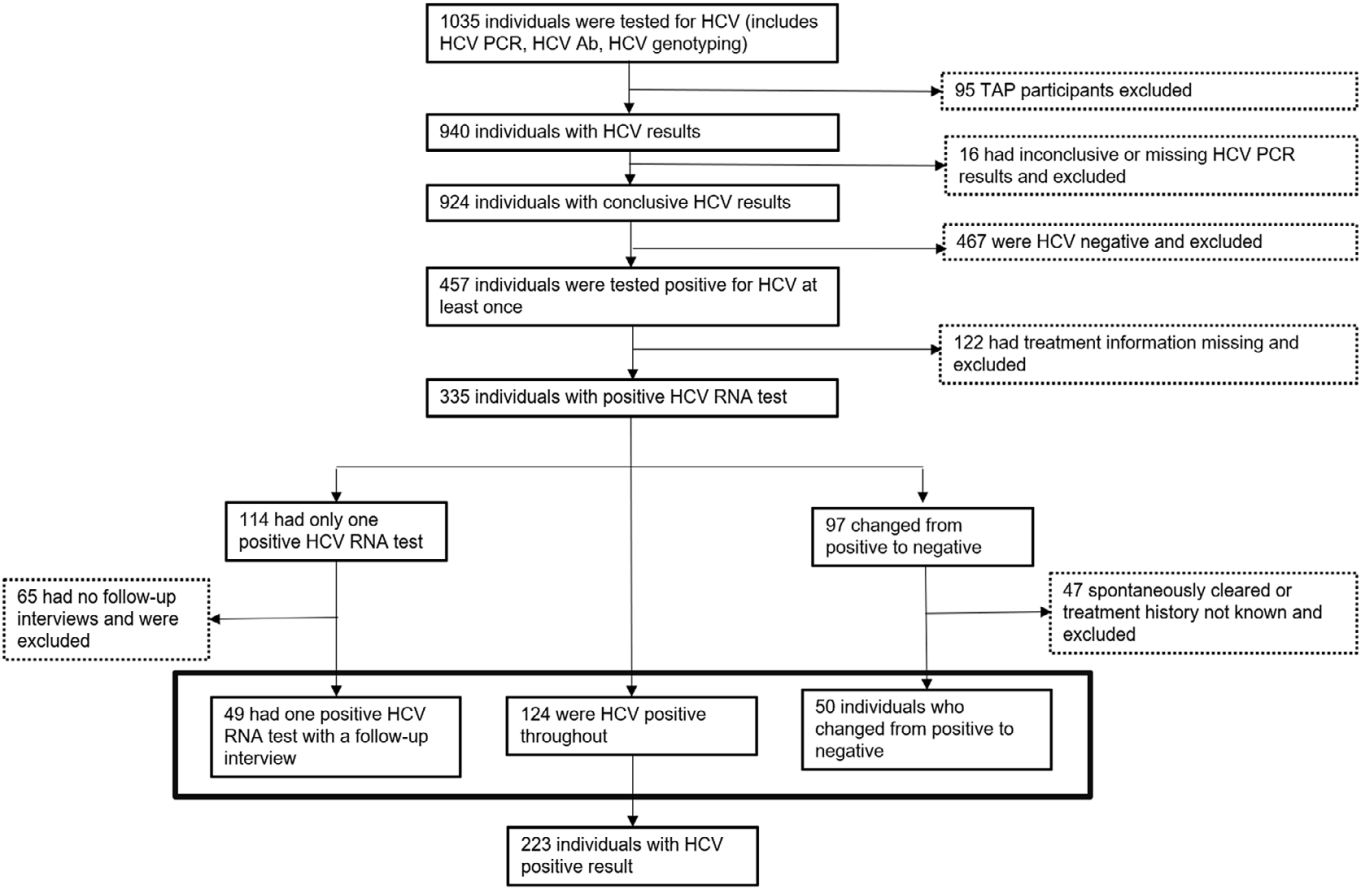
Schema of study population.

A total of 1035 participants had evidence of an HCV RNA test undertaken as part of the study protocol between November 2009 and March 2021. Of these participants, the following were excluded: 95 TAP participants; 16 participants with inconclusive or missing HCV RNA results; 467 participants who had negative HCV RNA results only; 122 participants without treatment information; 65 participants without a follow-up interview; and 47 participants who either spontaneously cleared or whose treatment history was uncertain (Figure 2). The final study population consisted of 223 participants who had an HCV RNA–positive result at least once with a follow-up visit during the study period.

There was an average of one year between the treatment and the interview from which the covariates were derived. All participants except one had an interval of fewer than four years between the interview and treatment dates (one individual had a 7.1 years’ gap between the two dates). The influence of this outlier was tested with minimal effect on outcomes observed, and therefore the outlier was included in the analysis.

### Demographic, social and drug use characteristics

Of 223 participants included in the analysis, 70.0% of the participants were male. The participants were born primarily in Australia and spoke English as their first language. Most participants reported being unemployed and almost half reported earning less than $AUD400 a week. Housing instability was prevalent among the participants, with almost half reporting sleeping rough, or living in a shelter or a hostel.

The median age of first injecting was 17 and the median injecting duration was 18 years. Most participants reported injecting drug use in the last month and 33.6% of the participants reported a history of incarceration. The detailed characteristics of the participants are reported in Table 1.

### Treatment initiation and time-to-treatment

**Figure 3.**
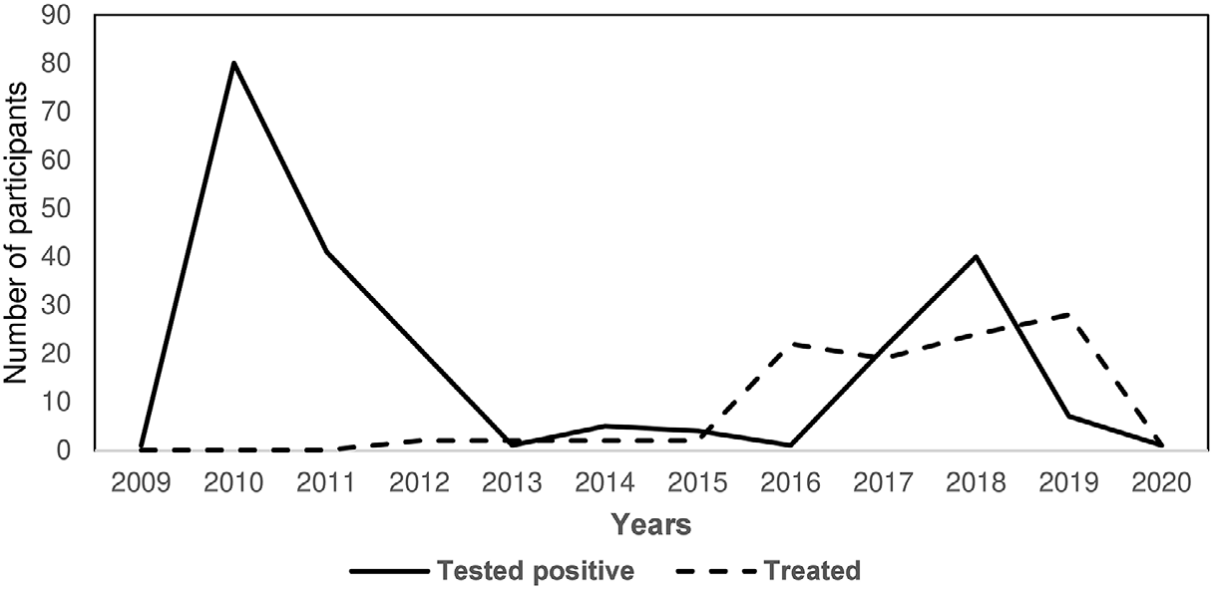
HCV treatment uptake and HCV first positive test date. Note: The peak in 2010 represents the initial recruitment period during which the participants had their first HCV RNA positive dates recorded.

Of the 223 participants included, 102 people (45.7%) reported treatment initiation. The number of participants testing positive increased rapidly as testing in the cohort commenced and then again with the second wave of recruitment in 2017 (Figure 3). Treatment uptake peaked in 2016 but declined steadily over subsequent years. Of the 121 people who did not report treatment initiation, 90 participants (70% of those not treated) were identified as potential missed opportunities for treatment.

**Figure 4.**
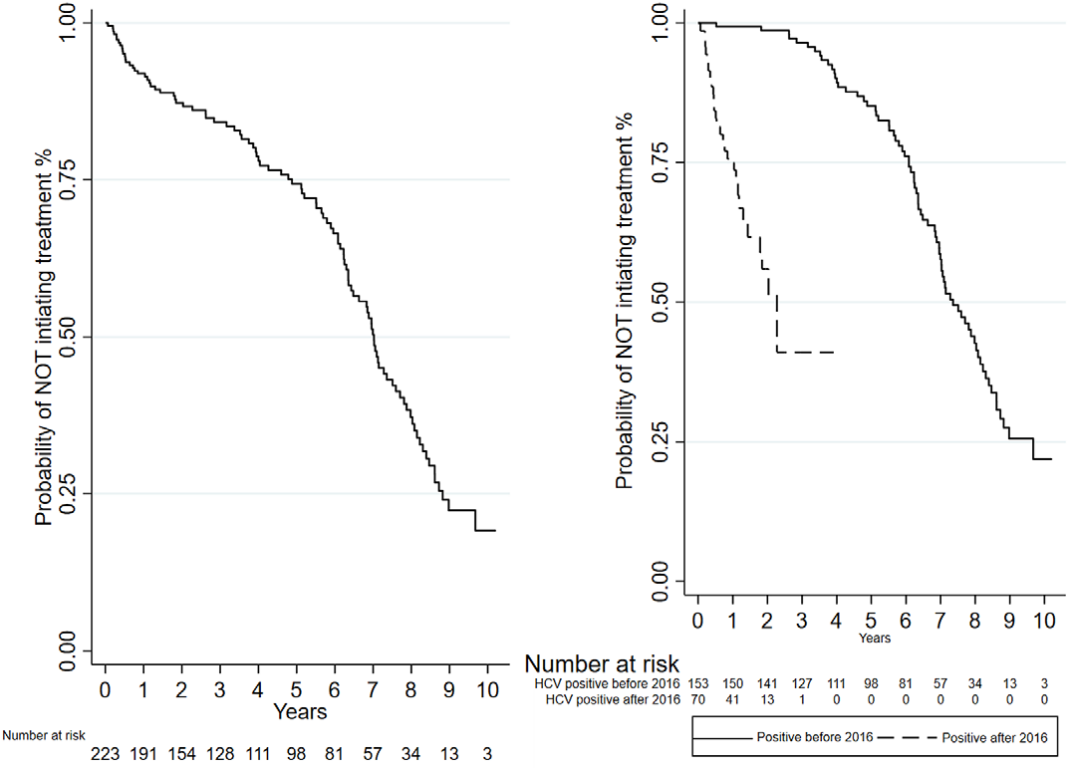
Kaplan-Meier curve for time-to-treatment initiation. 3a. Graph on the left represents overall time-to-treatment; 3b. graph on the right represents time-to-treatment by the date of the first positive HCV RNA test.

The overall median time-to-treatment from the first HCV RNA–positive test was 7.0 years (Figure 4a). In the subgroup analysis of individuals who tested positive after 2016 (n = 70), median time-to-treatment was reduced to 2.3 years (Figure 4b).

### Accelerated failure time model

The bivariate AFT analysis showed that a longer duration of injecting (TR 1.4, 95% CI 1.1–1.9) was associated with a longer time to initiate treatment, while engagement with health or social services (TR 0.6, 95% CI 0.4–0.9) and having a first positive HCV RNA test after March 2016 (TR 0.3, 95% CI 0.2–0.3) were associated with shortened time-to-treatment initiation (Table 1).

In the multivariate Weibull AFT analysis, treatment with OAT (TR 0.7, 95% CI 0.6–0.9), engagement with health or social services (TR 0.7, 95% CI 0.6–0.9), and having a first positive HCV RNA test after March 2016 (TR 0.3, 95% CI 0.2–0.3) were significant factors associated with shortened time-to-treatment initiation (Table 3). The median time-to-treatment was shortened by 30% if an individual was on OAT and engaged in health services, and by 70% if HCV tested positive after March 2016.

**Table 3.** Multivariate Weibull accelerated regression analysis for time-to-treatment initiation

Variables	Time ratio (TR)	95% CI	*P*-value
*Age at treatment (years)*			
20–30	Ref		
31–40	1.1	0.8–1.5	0.647
41–57	1.1	0.8–1.7	0.544
*Sex*			
Male	Ref		
Female	1.2	1.0–1.5	0.124
*Employment*			
Unemployed	Ref		
Employed	0.9	0.7–1.1	0.345
*Accommodation*			
Stable	1.1	0.9–1.3	0.552
Unstable	Ref		
*Social support*			
Yes	1.2	0.9–1.5	0.208
No	Ref		
*Duration of injecting drug use (years)*			
<18	Ref		
> = 18	1.1	0.9–1.4	0.315
*OAT in the last one month*			
Yes	**0.7**	**0.6–0.9**	**0.002**
No	Ref		
*Health or social service attendance in the last one month*			
Yes	**0.7**	**0.6–0.9**	**0.002**
No	Ref		
*First HCV RNA–positive date*			
Before 2016	Ref		
After 2016	**0.3**	**0.2–0.3**	**<0.001**

Note: Statistically significant values were highlighted in bold.

## Discussion

Our study found that shorter time-to-treatment initiation was associated with service engagement, including accessing OAT, and testing positive after 2016 when DAAs were made broadly available. However, even in the era of broad DAA access within a universal healthcare system, there were considerable delays between a positive HCV RNA test and treatment initiation, with a median of 2.3 years from the first positive HCV test to treatment. The overall treatment uptake also remained low among people who inject drugs, with only 45.7% of participants reporting treatment initiation over the follow-up period and 70% of those not treated representing missed opportunities. Consistent with broader trends in uptake of HCV treatment in Australia [8], our study also showed that treatment numbers peaked around 2016, followed by a gradual decline over recent years.

Our study finding of 2.3 years to treatment initiation if diagnosed after 2016 (and 7.0 years for the overall study cohort) showed that greater effort is required to ensure people diagnosed are supported to seek treatment early. Whilst there is no consensus on what constitutes an acceptable time-to-treatment initiation for HCV globally, treating early following infection not only benefits the individual but may also contribute towards preventing HCV at a population level [16, 21]. While findings suggest a need to strengthen referral pathways to care and treatment for hepatitis C after diagnosis, these delays occurred even in the context of facilitated referral attempts that occurred as part of the study protocol. These findings underscore the urgent need to attend to broader social and structural barriers to HCV treatment [24–26], including through the development of person-centred models of care and the implementation of adapted service models that facilitate timely (e.g., point-of-care testing and treatment at diagnosis) and convenient (e.g., integrated with other services targeting people who use drugs) treatment services [27, 28].

In our study, engagement in health and/or social services as well as recent OAT were associated with earlier treatment initiation. These findings are consistent with previous studies which show that people receiving OAT often have regular contact with drug treatment clinics or community health centres that can provide integrated one-stop models for HCV care, therefore increasing the chance of treatment uptake [29–31]. On the other hand, the treatment number remained low in our study population, with around 70% of those not treated representing ‘missed opportunities’ for treatment. A recent Australian study of 10 clinical services that prescribed OAT found that HCV testing was low (17%) in the first 12 months following OAT initiation among individuals prescribed OAT, even after DAAs were made broadly available [14]. Other studies have also reported that limited knowledge, lack of availability, and stigmatising attitudes within services can limit access to DAAs and have an impact on retention of care for people who inject drugs [23, 26, 29, 32, 33]. Whilst it is unclear why people (including our SuperMIX study participants) did not initiate treatment; it is likely that a combination of individual- and health system-level factors continue to present barriers to HCV care and that transformation within health systems is needed. Pedrana et al. [10] recommended strategies that include integrating HCV testing and treatment into alcohol and other drug, mental health, and housing services; providing financial incentives for treatment as currently used to incentivise childhood immunisation; using peers to help engage people in health care; and simplifying clinical pathways.

Our study was limited first by its reliance on self-reported data, which may introduce recall and/or social desirability. While such inaccuracies can be problematic in longitudinal studies, the validity of data collected in this study is enhanced by having highly trained researchers and established protocols and standardised methods. Furthermore, retrospective self-reports have been used widely in alcohol and other drug research and are deemed reliable [34, 35], and being treated or not for HCV, as the primary study outcome, is highly likely to be accurately recalled. **Secondly**, the time between follow-up visits meant some discordance in time between covariates and outcome measurements for some participants. However, the mean duration between the preceding interview and the treatment date was one year, and covariates used in the analysis were therefore likely to be broadly representative of participant experiences close to the treatment initiation. **Thirdly**, we excluded participants who showed evidence of resolved infection but without documented treatment. A small group of these participants may have been treated, underestimating the true number of treated participants. In addition, our complete case analysis approach meant that 2.2% of eligible cases were not included in our main analyses. As this is only a small percentage of the final cohort, any bias introduced by our approach was considered likely to be small. **Fourthly**, the analysis did not consider any variation in the time to relay the diagnosis among the participants, as these data were not collected. Lastly, the limited data precluded analysis of re-infection, and as a result, it was uncertain whether re-infection had any effect on the rate of treatment initiation.

## Conclusion

Our study demonstrated that the timely treatment of HCV infection among people who inject drugs remains challenging, even in the context of widespread access to DAA therapies. The study findings showed shortened time-to-treatment with higher levels of contact with services and OAT. However, missed opportunities continue to present even among those who are in regular contact with these services. These challenges suggest new approaches within the health system may be needed, including integration of HCV care into routine services, improving clinical pathways and linkage between services, and adopting tailored approaches to meet the specific needs of people who inject drugs.

## Data Availability

The aggregated data that support the findings of this study are available from the corresponding author, P Aung, upon reasonable request. The data requests are subject to approval by the chief investigators and the working group.

## Supplementary material

For supplementary material accompanying this paper visit http://doi.org/10.1017/S0950268823000675. click here to view supplementary material
