# Supplementary material for: Time-to-hepatitis C treatment initiation among people who inject drugs in Melbourne, Australia
Source: Epidemiol Infect. 2023 May 9;151:e84. doi: 10.1017/S0950268823000675 (PMC10226186; doi:10.1017/S0950268823000675)
Supplement: Supplementary file 1 [file hygsup.zip › S0950268823000675sup001.docx]

**Supplementary Material**

Journal: Epidemiology and Infection

Title: Time-to-hepatitis C treatment initiation among people who inject drugs in Melbourne, Australia

Authors: Phyo TZ. Aung, Tim Spelman, Anna L. Wilkinson, Paul M. Dietze, Mark A. Stoové, Margaret E. Hellard

**Appendix 1**

The individual variables studied are

1. Demographic characteristics: age at the time of treatment initiation or at the time of interview(derived from treatment initiation date and the interview date), sex, country of birth (Australia or other), language spoken at home (English, other), and Aboriginal and Torres Strait Islander status.
2. Socioeconomic characteristics: highest level of education (< year 10, year 10-11, year 12 or higher), employment, weekly income (<AUD$400, ≥AUD$400), housing, social support, incarceration in the 12 months prior to interview. Our study participants reported employment in the form of full time or part-time employment, full-time or part-time studies or engaged in home duties. Housing was recorded as stable if participants reported their residence as their own home, a rental property or their parents’ home or if self-reported stable accommodation. On the other hand, accommodation was considered unstable if the participant reported living in a boarding house, a hotel, a shelter, a caravan park or reported sleeping rough. Social support was considered to be present if the participant reported social support from any of the following: partners, family members, relatives, neighbors, friends, coworkers classmates, religious groups or social support from health professionals such as health care workers, counsellors, therapists, medical doctors, or other.
3. Drug use characteristics: drug related variables were dichotomized according to their median values. The characteristics analysed are age of first injected drugs (below median age, above median age), duration of injection career (calculated by subtracting age of first injecting drugs from age at the time of treatment initiation), and injecting drug use within the past month (included if the participant reported injecting any of the following substances: heroine, methadone, suboxone, buprenorphine, morphine, oxycodone, other opioids, benzodiazepines, amphetamine, cocaine, antipsychotics, antidepressants, pregabalin and doxylamine).
4. Health and social service use: overall health service use in the last month was generated if the participant reported attending or interaction with any of the following services which include emergency department, outpatient, inpatient, primary care service with support for injecting drug use, general practice, specialist service, dentist, allied health , ambulance, mental health professional, social/social worker, drug counselling, job service, unemployment service, parole officer, and child protection worker. Current opioid agonist therapy (OAT) was recorded if, at the time of interview, use of methadone, suboxone, buprenorphine and naltrexone was reported.
