# Supplementary material for: Time-to-hepatitis C treatment initiation among people who inject drugs in Melbourne, Australia
Source: Epidemiol Infect. 2023 May 9;151:e84. doi: 10.1017/S0950268823000675 (PMC10226186; doi:10.1017/S0950268823000675)
Supplement: Supplementary file 1 [file hygsup.zip › S0950268823000675sup002.docx]

**Supplementary Material**

Journal: Epidemiology and Infection

Title: Time-to-hepatitis C treatment initiation among people who inject drugs in Melbourne, Australia

Authors: Phyo TZ. Aung, Tim Spelman, Anna L. Wilkinson, Paul M. Dietze, Mark A. Stoové, Margaret E. Hellard

The table below represents a sensitivity analysis that includes the year of diagnosis as a covariate. In this analysis, every additional year of diagnosis was associated with a 20% reduction (TR 0.8) in the rate of treatment uptake. On average, participants who had a longer disease duration were more likely to be treated during the study period, regardless of whether they were diagnosed before or after 2016.

**Supplementary Table S1: Multivariate Weibull accelerated regression analysis for time-to-treatment initiation, including year of diagnosis as a covariate**

| **Variables** | **Time ratio (TR)** | **95% CI** | **p-value** |
| --- | --- | --- | --- |
| **Age at treatment (years)** |  |  |  |
| 20 – 30 | Ref |  |  |
| 31 – 40 | 1.1 | 0.8 – 1.5 | 0.457 |
| 41 – 57 | 1.2 | 0.8 – 1.7 | 0.488 |
|  |  |  |  |
| **Sex** |  |  |  |
| Male | Ref |  |  |
| Female | 1.2 | 1.0 – 1.4 | 0.137 |
|  |  |  |  |
| **Employment** |  |  |  |
| Unemployed | Ref |  |  |
| Employed | 0.8 | 0.7 – 1.0 | 0.104 |
|  |  |  |  |
| **Accommodation** |  |  |  |
| Stable | 1.1 | 0.9 – 1.3 | 0.503 |
| Unstable | Ref |  |  |
|  |  |  |  |
| **Social support** |  |  |  |
| Yes | 1.1 | 0.8 – 1.4 | 0.560 |
| No | **Ref** |  |  |
|  |  |  |  |
| **Duration of injecting drug use (years)** |  |  |  |
| <18 | Ref |  |  |
| >=18 | 1.1 | 0.9 – 1.4 | 0.252 |
|  |  |  |  |
| **OAT in the last one month** |  |  |  |
| Yes | **0.8** | **0.7 – 0.9** | **0.036** |
| No | Ref |  |  |
|  |  |  |  |
| **Health or social service attendance in the last one month** |  |  |  |
| Yes | **0.7** | **0.6 – 0.9** | **0.005** |
| No | Ref |  |  |
|  |  |  |  |
| **Year of diagnosis** |  |  |  |
| Year of diagnosis | **0.8** | **0.8 – 0.9** | **<0.001** |
